# Bovipain-2, the falcipain-2 ortholog, is expressed in intraerythrocytic stages of the tick-transmitted hemoparasite *Babesia bovis*

**DOI:** 10.1186/1756-3305-3-113

**Published:** 2010-11-23

**Authors:** María Mesplet, Ignacio Echaide, Mariana Dominguez, Juan J Mosqueda, Carlos E Suarez, Leonhard Schnittger, Monica Florin-Christensen

**Affiliations:** 1 Instituto de Patobiología, Centro de Investigaciones en Ciencias Veterinarias y Agronómicas, Instituto Nacional de Tecnología Agropecuaria, INTA-Castelar, Argentina; 2 Estación Experimental Agropecuaria, INTA-Rafaela, Argentina; 3 Universidad Autónoma de Querétaro, Campus Juriquilla, México; 4 Animal Disease Research Unit-USDA-ARS, Pullman, WA, USA; 5 Consejo Nacional de Investigaciones Científicas y Técnicas (CONICET), Buenos Aires, Argentina

## Abstract

**Background:**

Cysteine proteases have been shown to be highly relevant for Apicomplexan parasites. In the case of *Babesia bovis*, a tick-transmitted hemoparasite of cattle, inhibitors of these enzymes were shown to hamper intraerythrocytic replication of the parasite, underscoring their importance for survival.

**Results:**

Four papain-like cysteine proteases were found to be encoded by the *B. bovis *genome using the MEROPS database. One of them, the ortholog of *Plasmodium falciparum *falcipain-2, here named bovipain-2, was further characterized. Bovipain-2 is encoded in *B. bovis *chromosome 4 by an ORF of 1.3 kb, has a predicted molecular weight of 42 kDa, and is hydrophilic with the exception of a transmembrane region. It has orthologs in several other apicomplexans, and its predicted amino acid sequence shows a high degree of conservation among several *B. bovis *isolates from North and South America. Synteny studies demonstrated that the *bovipain-2 *gene has expanded in the genomes of two related piroplasmids, *Theileria parva *and *T. annulata*, into families of 6 and 7 clustered genes respectively. The *bovipain-2 g*ene is transcribed in *in vitro *cultured intra-erythrocyte forms of a virulent and an attenuated *B. bovis *strain from Argentina, and has no introns, as shown by RT-PCR followed by sequencing. Antibodies against a recombinant form of bovipain-2 recognized two parasite protein bands of 34 and 26 kDa, which coincide with the predicted sizes of the pro-peptidase and mature peptidase, respectively. Immunofluorescence studies showed an intracellular localization of bovipain-2 in the middle-rear region of *in vitro *cultured merozoites, as well as diffused in the cytoplasm of infected erythrocytes. Anti-bovipain-2 antibodies also reacted with *B. bigemina*-infected erythrocytes giving a similar pattern, which suggests cross-reactivity among these species. Antibodies in sera of two out of six *B. bovis*-experimentally infected bovines tested, reacted specifically with recombinant bovipain-2 in immunoblots, thus demonstrating expression and immunogenicity during bovine-infecting stages.

**Conclusions:**

Overall, we present the characterization of bovipain-2 and demonstrate its *in vitro *and *in vivo *expression in virulent and attenuated strains. Given the involvement of apicomplexan cysteine proteases in essential parasite functions, bovipain-2 constitutes a new vaccine candidate and potential drug target for bovine babesiosis.

## Background

The tick-transmitted apicomplexan hemoprotozoon *Babesia bovis *continues to impose serious limitations to cattle development worldwide [1,2]. A better understanding of its pathogenic mechanisms and the exploitation of the recently sequenced genome [3] is needed for the identification of molecules that are involved in the host-pathogen and vector-pathogen interface, which can lead to improved control strategies against this parasite.

The search for relevant parasite molecules can benefit from the information available for *Plasmodium falciparum*, another arthropod vector-transmitted apicomplexan protozoon with an intraerythrocytic life stage, that shares pathogenicity mechanisms with *B. bovis *[4]. Plasmodial peptidases have been shown to play vital functional roles and have been proposed as vaccine candidates [5]. The best characterized *P. falciparum *peptidases are the falcipains, which are cysteine peptidases that belong to clan CA, subfamily C1A.

Assortment of peptidases into clans is based on the presence of sequence motifs around the catalytic residues, their evolutionary relationships and/or similarities in their tertiary structure. Clans, in turn, are subdivided in families, according to their amino acid sequence similarities. Cysteine peptidases of clan CA utilize catalytic glutamine, cysteine, histidine and asparagine residues that are invariably in this order [6]. These four amino acids are present in three separate, well conserved regions of the primary sequence that correspond to the mature protease, which are known as the eukaryotic thiol (cysteine) proteases cysteine, histidine, and asparagine active site regions.

Falcipain-2 has shown to be involved in digestion of host erythrocyte hemoglobin in the parasite food vacuole [7,8]. The amino acids that result from this process are used for parasite protein synthesis [9,10], and contribute to maintain the osmotic stability of the parasite [11]. Hemoglobin degradation might provide space for the growth of the parasite inside the erythrocyte [12]. Additionally, falcipain-2 has been shown to cleave host erythrocyte membrane skeletal proteins ankyrin and protein 4.1. The removal of the carboxyl terminus of ankyrin weakens its interaction with the erythrocyte membrane and yields an increased rate of membrane fragmentation of infected erythrocytes [13]. In addition, falcipain-2 cleaves protein 4.1 within a region of the spectrin-actin binding domain critical for erythrocyte membrane stability [14]. It has been postulated that the proteinase-induced ankyrin and protein 4.1 degradation destabilizes the erythrocyte membrane skeleton, which in turn facilitates parasite release [15]. Furthermore, it has been shown that cysteine peptidases might be involved in the differentiation of plasmodial gametocytes into ookinetes. Torres et al [16] demonstrated that serine/cysteine protease inhibitors TPCK and TLCK, but not the serine protease specific inhibitors PMSF and leupeptin, inhibited exflagellation centers formation, suggesting a participation of cysteine proteases in *P. berghei *gamete activation and zygote development.

Cysteine proteinases have been shown to play critical roles in the pathogenicity of other parasitic protozoans as well. Thus, they have been identified as virulence factors in *Leishmania amazonensis *and *Entamoeba histolytica *[17,18]. Virulence is intimately associated with proteolysis and invasion of cells and/or tissues by intracellular and extracellular parasites [19]. Accordingly, cysteine proteinases of *Toxoplasma gondii *and *Trypansoma cruzi *have been found to be involved in host cell and tissue invasion and egress [18,20]. Adherence of *E. histolytica *to erythrocytes and epithelial cells as well as excystation processes of *Cryptosporidium *sp. and *Giardia *sp. [18] have been shown to be mediated by parasite cysteine proteinases.

This type of enzyme is ubiquitous in all kingdoms of organisms. In mammals, cysteine proteinases function in diverse processes like apoptosis, prohormone processing, tissue remodelling, turnover of the extracellular matrix, and extracellular degradation of foreign proteins [21,22]. Importantly, lysosomal cysteine peptidases also regulate the immune response by mediating antigen presentation of classical MHC class II and non-classical MHC class-I molecule CD1D [23].

Cysteine peptidases have so far not been characterized in *B. bovis*. However, evidence of their importance for the survival of these parasites was obtained by the observation that specific inhibitors of these enzymes impaired merozoite growth *in vitro *[24]. As in *P. falciparum*, cysteine peptidases may be involved in the erythrocyte egress of *B. bovis *merozoites, a prerequisite for the maintenance of the asexual propagation of the parasite, and/or nutrition of the trophozoite and merozoite parasite stages that reside within the erythrocyte through degradation of hemoglobin. Additionally, *B. bovis *cysteine peptidases may also play an important role in several differentiation steps of the parasite (sporozoite, trophozoite, merozoite, kinete) as well as in invasion and evasion processes within the tick vector tissues.

The aim of this work has been to identify and characterize the falcipain-2 ortholog in *B. bovis*, as well as to make an inventory of cysteine proteinases in this parasite and two related piroplasmids.

## Results

### Identification of putative cysteine peptidases of the C1 family in the *B. bovis *T2Bo genome

Four putative cysteine peptidases of clan CA, subfamily C1A [6], were identified in the *B. bovis *T2Bo predicted proteome. Two of these cysteine proteinases belong to the family of dipeptidylpeptidase I of the *Plasmodium*-type [MEROPS:C01.124] which is confined to protozoans. This type of cysteine proteinases is composed of the cathepsin C exclusion domain, whose function is to prevent the approach of a polypeptide apart from its termini, and the catalytically active cysteine peptidase region. The remaining two cysteine proteinases were found to be papain homologues of the *Theileria*-type. This type of proteases are synthesized as inactive proenzymes and proteolytic cleavage of the inhibitor region [MEROPS:I29.UPW] is required for activation of the peptidase C1 unit [MEROPS:C01.079]. Apart from protozoans, this type of papain-like cysteine proteinases has also been found in other eukaryotes. The identification number, chromosome location, current annotation status, predicted protein size and active site region localization of these four cysteine peptidases are shown in Table 1.

**Table 1:** Babesia bovis cysteine peptidase C1 family

GenBank Id	Annotation	Chr	Predicted protein length (aa)	Catalytic region localization	MEROPS annotation of catalytic region
XP_001610695	**cysteine protease 2**	**4**	**445**	**235-444**	**papain homolog (Theileria type) (C01.079)**

XP_001612131	papain family cysteine peptidase	3	435	135-426	papain homolog (Theileria type) (C01.079)

XP_001609546	cathepsin C precursor	2	530	261-511	dipeptidylpeptidase I (Plasmodium type) (CO1.124)

XP_001608716	cathepsin C precursor	1	546	279-530	dipeptidylpeptidase I (Plasmodium type) (CO1.124)

All putative cysteine peptidases were identified in the *B. bovis *T2B predicted proteome using the MEROPS database. Protein identification number, current Genbank annotation, chromosome (chr), predicted protein length, catalytic protein localization, and MEROPS annotation of the catalytic region of those putative proteins belonging to the C1 family are shown.

### Molecular characterization of bovipain-2

Among the four predicted cysteine proteases mentioned above, the peptide XP_001610695 showed to be orthologous to *P. falciparum *falcipain-2. According to current nomenclature standards, this protein is referred to as bovipain-2 in this work. Bovipain-2 orthologs are present in *B. bigemina*, *T. annulata*, *T. parva*, *P. knowlesi*, and *P. vivax *(Table 2). A very high E value was also found with a cysteine peptidase of *T. orientalis *(E value, 7e-73) and *B. equi *also known as *T. equi *[25] (E value, 3e-70), however, as the complete genome sequence of these two organisms is not available, orthology could not be established.

**Table 2:** Babesia bovis bovipain-2-related proteins in other apicomplexan organisms

GenBank Id	Organism	Annotation	% identity	Bovipain-2 ortholog	**Ref**.
ACO94081	*Babesia bigemina*	babesipain-1	48.5	Yes	1

BAD08222	*Theileria orientalis*	cysteine peptidase 2	36.6	NA	-

XP_954970	*T. annulata*	cysteine peptidase precursor, tacP	36.6	Yes	2

XP_763298	*T. parva*	cysteine proteinase	36.5	Yes	3

AAC17994	*B. equi*	cysteine peptidase	35.0	NA	4

XP_001615274	*Plasmodium vivax*	vivapain-2	29.3	Yes	-

XP_002259153	*P. knowlesi*	falcipain ortholog	28.8	Yes	5

XP_001347832	*P. falciparum*	falcipain-2B	28.5	Yes	6

Proteins with the highest scores of identity with bovipain-2 amino acid sequence (T2B strain) are shown. A Blosum 62 matrix was used to calculate identity percentages. Orthology was established by reverse BLAST against available genome sequences of corresponding organisms. NA: not applicable as the complete genome sequence was not available. References (Ref.) are as follows: (1) [35]; (2) [57]; (3) [58]; (4) [59]; (5) [26]; (6) [60].

**Figure 1:**
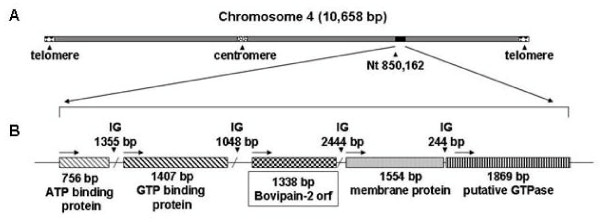
**Localization of the bovipain-2 gene in the genome of the *Babesia bovis *strain T2Bo**. A. Schematic representation of chromosome 4 of the *B. bovis *strain T2Bo showing the relative localization of the bovipain-2 encoding gene. The localization of the centromere and telomeres are indicated by arrows. B. Organization of the *B. bovis *~39.4 kbp genomic region containing the locus of bovipain-2. The orientation of the open reading frames (ORF) is shown with black arrows. IG: intergenic regions.

The bovipain-2 encoding gene is located half way between the centromere and the 3' telomere of chromosome 4. It is surrounded, upstream, by a gene encoding a GTP-binding protein and, downstream, by a gene encoding a hypothetical membrane protein (Figure 1). No other peptidase-encoding genes are found in close vicinity. The *bovipain-2 *ORF is 1338 bp long, and encodes for a putative protein with a calculated molecular weight of 42 kDa.

**Figure 2:**
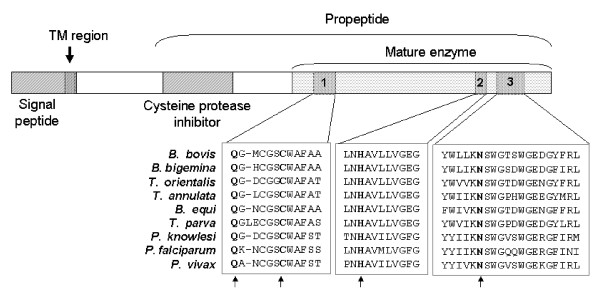
**Domain prediction of bovipain-2**. Putative functional domains in bovipain-2 were predicted based on the amino acid sequence of the T2B strain. The numbers indicate the eukaryotic thiol (cysteine) proteases cysteine (1); histidine (2) and asparagine (3) catalytic regions. Amino acids in bold represent active sites (Q254, C260, H389, N411). Sequence alignments of these three regions in several *Babesia*, *Theileria *and *Plasmodium *spp. are shown. Arrows indicate amino acids of the active site.

Secondary structure prediction of the bovipain-2 ORF showed three distinct regions: an N-terminal hydrophilic region of 43 amino acids (aa), predicted to be located in an aqueous compartment at one side of a membrane, a 22 aa-long hydrophobic transmembrane domain, and a 380 aa-long region at the opposite side of the membrane that extends to the C-terminus. Domain predictions (Figure 2) showed the typical prepropeptide organization of cysteine proteases of the papain family, including a 43 aa-long signal peptide, a 24 aa transmembrane domain, a spacer, and a 320 aa-long propeptide (which corresponds to a predicted molecular weight of 34 kDa). This propeptide is composed of a cysteine peptidase inhibitor, a spacer and a 209 aa-long mature enzyme of an estimated molecular weight of 26 kDa.

Post-translational modification of 3 sites that are potentially N-glycosylated (N113, N198, and N221) and 1 site that is potentially O-glycosylated (T204) could be predicted based on sequons in the propeptide/spacer region of bovipain-2. As the available bioinformatic tools have been developed for mammalian organisms, the validity of these predictions in *Babesia *needs to be verified by direct experimental evidence.

The mature enzyme owns three stretches of amino acids that contain the catalytic residues (Q254, C260, H389, and N411). Upon folding, these amino acid sequences configure the 3D structure of the catalytic center of the proteinase. These three stretches are known as 1) eukaryotic thiol (cysteine) proteases cysteine (aa 254 to 265), 2) histidine (aa 387 to 397), and 3) asparagine (aa 406 to 425) active site regions, since these residues are essential for enzymatic activity. Their sequence is highly conserved among several apicomplexan parasites, as shown in Figure 2.

Bovipain-2 predicted amino acid sequences encoded in the genome of different *B. bovis *geographic isolates from USA, Argentina, Uruguay and Brazil and Mexico were aligned and compared (data not shown). A high percentage of overall identity (97.8%) was observed among these sequences, with polymorphism in 10 sites (Table 3). All ten of these amino acid substitutions are conservative, and were *in silico *predicted not to affect protein function, as the exchanged amino acids have similar physico-chemical characteristics.

**Table 3:** Strain polymorphism of bovipain-2

Strain/isolate	GenBank Id	Position of polymorphic amino acid residue									
		71	76	*89*	*121*	*139*	*145*	*201*	*365*	*401*	*420*

T2Bo	BBOV_IV007730	S	S	N	V	**T**	T	V	A	**A**	D

BboR1A	GQ412131	S	S	N	V	S	**S**	**I**	**G**	**A**	D

BboS2P	GQ412136	**G**	**A**	**S**	**I**	S	**S**	V	A	T	D

BboM3P	GQ412133	S	S	N	V	**T**	T	V	A	T	D

Brazil	GQ412132	S	S	**S**	V	S	T	**I**	A	T	**E**

Uruguay	GQ412134	S	S	**S**	V	S	T	**I**	**G**	**A**	D

Veracruz	GQ412135	S	S	N	V	**T**	T	V	A	T	D

Common physico-chemical characteristics		s	s	p;s	a	p;s	p;s	a	s;h	s;h	n

The complete bovipain-2 coding sequence of different isolates was sequenced. The table shows the polymorphic sites and the amino acid substitutions, after alignment of predicted peptide sequences. Amino acid polymorphic positions within the active site region are in italics. The least frequent substitution in each column is in bold. Common physico-chemical characteristics of amino acids found at each polymorphic site are depicted in the last row: p: polar; a: aliphatic; h: hydrophobic; s: small or tiny; n: negatively charged. Single-letter amino acids code was used.

### Synteny and phylogenetic studies with *Theileria annulata *and *T. parva *cysteine peptidases

**Figure 3:**
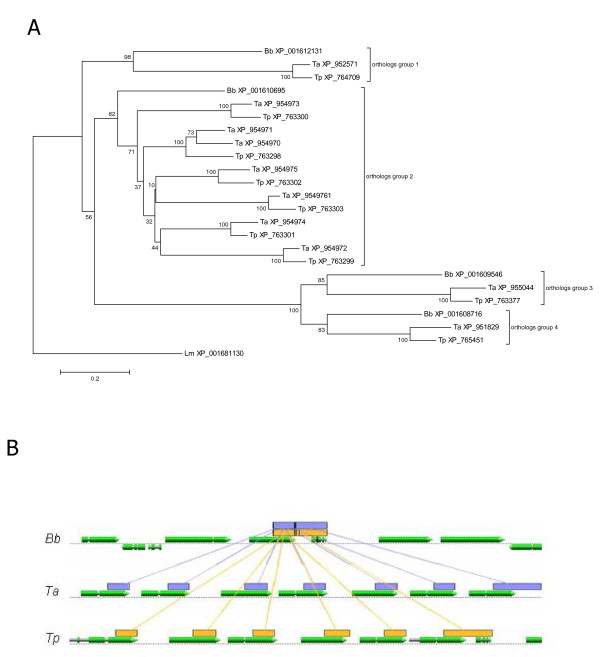
**Phylogenetic and synteny relationships between *B. bovis*, *T. annulata *and *T. parva *C1 cysteine peptidases**. A. Phylogenetic relationships of cysteine peptidases of *B. bovis *and their *Theileria annulata *and *T. parva *orthologs/paralogs as analyzed by neighbor joining. The tree is drawn to scale, with branch lengths in the same units as those of the evolutionary distances used to infer the phylogenetic tree. The evolutionary distances were computed using the Poisson correction method. Deletion of all positions containing gaps and missing data resulted in a total of 171 positions in the final dataset. The percentage of replicate trees in which the associated taxa clustered together in the bootstrap test (1000 replicates) is shown next to the branches. The GenBank reference sequence number of each protein is shown. Bb: *B. bovis*; Ta: *Theileria annulata*; Tp: *T. parva*; Lm: *Leishmania major *cathepsin L-like protein is used as outgroup. B. Synteny studies of the bovipain-2 encoding genome region with the ortholog/paralogs encoding regions of *T. annulata *and *T. parva*. Lines that connect the catalytic region of the bovipain-2 [GenBank: XP_001610695] encoding gene and the catalytic regions of related cysteine proteinases encoding genes of *T. annulata*, and *T. parva *represent high scoring sequence pairs (HSPs). Reference protein sequence numbers from right to left: *T. annulata*, [GenBank: XP_954970, XP_954971, XP_954972, XP_954973, XP_954974, XP_954975, XP_954976], and *T. parva*, [GenBank: XP_763298, XP_763298, XP_763298, XP_763298, XP_763298, XP_763298]. A small ORF encoding a hypothetical protein [GenBank XP_001610696] in *B. bovis *has corresponding HSPs in *T. annulata *and *T. parva*. In *T. annulata *this ORF has not been annotated.

The phylogenetic relationships of the four identified putative cysteine peptidases of *B. bovis *and related orthologs and/or paralogs of *T. annulata *and *T. parva *were, analyzed (Figure 3A). Clades 1, 3, and 4 each contain one *B. bovis *cysteine peptidase with their respective *T. annulata *and *T. parva *orthologous counterpart. Orthology of the protein sequences in clades 1, 3, and 4 were also confirmed by a reverse BLAST test. In contrast, clade 2, contains the *B. bovis *cysteine peptidase defined in this work as bovipain-2, as well as an evolutionary closely related family of 7 *T. annulata *and 6 *T. parva *cysteine proteinases (Figure 3B). Comparative genomics of the bovipain-2 coding region of *B. bovis *with corresponding cysteine proteinase encoding regions of *T. annulata*, and *T. parva *genomes demonstrated that the *bovipain-2 *gene has expanded into a gene family of 7 and 6 cysteine proteinase encoding genes, respectively. Both gene families are clustered and have possibly originated from several gene duplication events. It is noteworthy that a small ORF encoding a hypothetical protein [GenBank: XP_001610696] that is lying downstream of the bovipain-2 encoding gene in *B. bovis *has high scoring sequence pairs (HSPs) in *T. annulata *and *T. parva*. However, only in *T. parva*, but not in *T. annulata *this ORF has been annotated. This highly likely represents an example of a mis-annotation as this ORF has been reported to be conserved in apicomplexan parasites [26].

### Transcription and expression of bovipain-2 in *B. bovis in vitro *cultured merozoites

**Figure 4:**
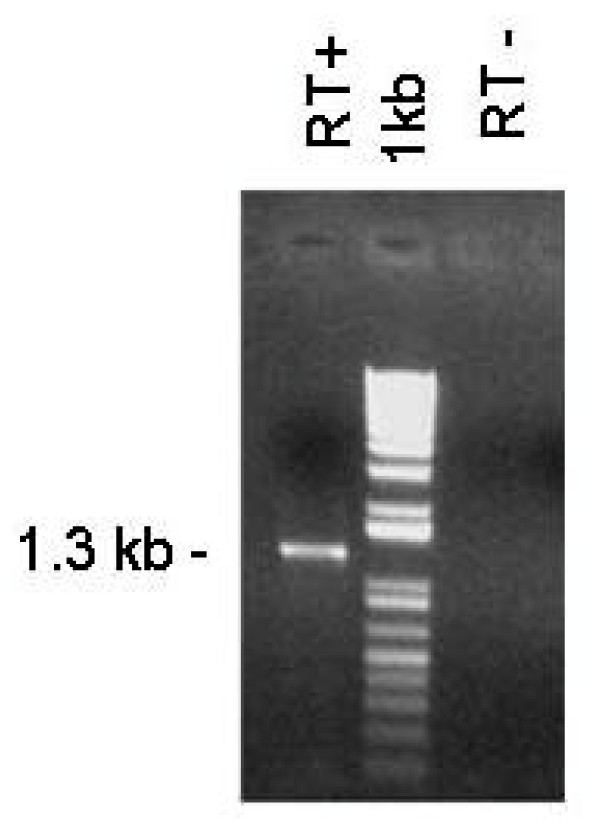
**Transcription of the bovipain-2 gene**. Bovipain-2 transcripts were detected by total RNA extraction of *in vitro *cultured *B. bovis *merozoites (BboS2P strain), followed by incubation with reverse transcriptase and PCR amplification of the complete ORF (RT+). To rule out DNA contamination, an equal amount of RNA was not incubated with reverse transcriptase and then treated as above (RT-). 1 kb: DNA size standard. The size of the obtained amplicon is marked at the left.

RT-PCR, using total RNA from *in vitro *cultured parasites from the Argentine virulent strain BboS2P (Figure 4) and the attenuated strain BboR1A (not shown) as template, demonstrated that the *bovipain-2 *gene is transcribed in intra-erythrocytic stages of both strains. Sequence comparison between the DNA and the resulting cDNA sequences showed the absence of introns.

**Figure 5:**
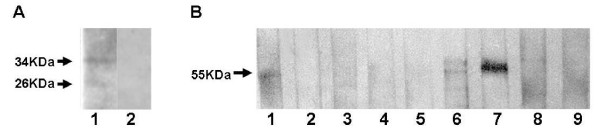
**Expression and immunogenicity of bovipain-2**. A. Expression of bovipain-2 in *in vitro *cultured merozoites. Aliquots of a merozoite suspension of the BboS2P strain of *B. bovis *were electrophoresed, blotted and exposed to antibodies against (1) recombinant bovipain-2, or (2) normal serum. Antibody binding was detected by incubation with peroxidase-labeled anti-mouse IgG and chemiluminiscence. Bands of 34 and 26 kDa correspond to the expected sizes of bovipain-2 pro-protein and mature protein, respectively. B. Immunogenicity of bovipain-2 in *B. bovis*-experimentally infected cattle. Stripes with blotted, partially purified bovipain-2 were incubated with sera from different bovines experimentally infected (day 63 p.i.) with the BboR1A strain (stripes 1-3) or the BboS2P strain of *B. bovis *(4-6); non infected bovine sera (8-9); or a monoclonal antibody that reacts with the histidine tag of the recombinant protein (7). A band of 50-55 kDa was recognized in samples 1, 6 and 7 by reaction with peroxidase-labeled species-specific secondary antibodies, followed by chemiluminiscence detection.

Antibodies against a recombinant form of bovipain-2 recognized at least two distinct proteins of *in vitro *cultured merozoites of the BboS2P strain in immunoblots. Two bands of 26 and 34 kDa were observed, which coincide with the expected sizes of the proenzyme and the mature enzyme, respectively (Figure 5A).

**Figure 6:**
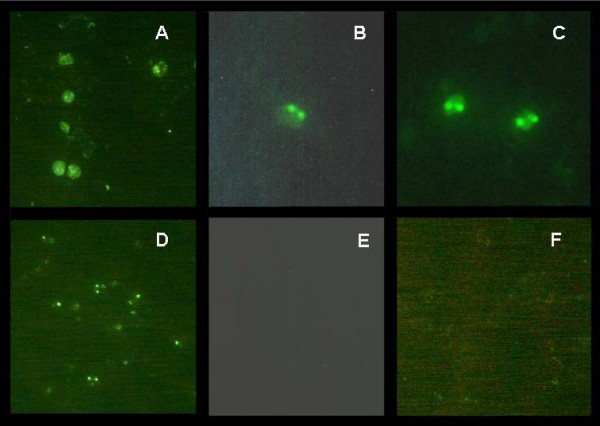
**Localization of bovipain-2 in merozoites**. Erythrocytes infected with *B. bovis *strain BboS2P (A, D, E); *B. bovis*, strain RAD (B); *B. bigemina*, Mexico strain (C), or non infected erythrocytes (F) were incubated with murine anti-bovipain-2 antibodies (A, B, C, F); anti-*B. bovis *RAP-1 monoclonal antibody 23/70.174 (D) or pre-immune murine serum (E); followed by detection with FITC- (A,D,E,F) or Alexa Fluor 488- (B,C) labeled anti-murine IgG, and observation by epifluorescene, with 400 × (A,D,E,F) or 1000 × magnification (B,C). Bovipain-2-associated fluorescence was observed as a round spot inside merozoites and within the erythrocyte cytoplasm (A,B,C), different from the punctuated fluorescence associated to RAP-1 (D). No cross-reactivity with erythrocyte proteins was detected (F); and no unspecific reaction was observed with pre-immune mouse serum (E).

Immunofluorescence confirmed the expression and intracellular localization of bovipain-2 in *in vitro *cultured merozoites from an Argentine (BboS2P) and a Mexican (RAD) strains (Figure 6A and 6B). Fluorescence microscopy analysis indicated that the signal appeared concentrated in a dense spot, in the middle-rear region of the cell (Figure 6B). Additionally, diffuse fluorescence was also observed in the cytoplasm of erythrocytes infected with merozoites of either strain, but not in non-infected erythrocytes (Figure 6F), thus demonstrating that the pattern of fluorescence observed was bovipain-2-specific and not due to possible cross-reaction with erythrocyte proteins. As expected [27], anti-rhoptry associated protein-1 (RAP-1) monoclonal antibodies tested on the same smears yielded a punctuated pattern, with no erythrocyte cytoplasm reaction (Figure 6D), and normal mice serum gave no signal (Figure 6E). *B. bigemina *merozoites of an Argentine (BbiS2P) (not shown) and a Mexican (Mexico strain) strain (Figure 6C) also reacted with anti-bovipain-2 antibodies, as shown by immunofluorescence, yielding a similar pattern to that observed for *B. bovis*. Thus, the immunofluorescence data indicates that bovipain-2 is expressed in intra erythrocytic stages of *B. bovis*.

### Antigenicity of bovipain-2 in *B. bovis*-infected cattle

Partially purified recombinant bovipain-2 was tested in immunoblots against serum samples from bovines experimentally infected with the BboS2P (n = 3) or the BboR1A (n = 3) strains of *B. bovis *(day 63 p.i.). Non *B. bovis*-reactive sera from two bovines from a tick-free region of Argentina were used as negative controls. Only one of the BboS2P-infected and one of the BboR1A-infected sera reacted with recombinant bovipain-2 (Figure 5B), while all BboS2P and BboR1A sera strongly reacted with recombinant MSA-2c (not shown), a previously characterized immunodominant protein of *B. bovis *[28]. These results indicate that bovipain-2 is expressed and immunogenic in the course of bovine infections with these two strains.

## Discussion

*P. falciparum *falcipain-2 has received a great amount of attention as a target for therapeutic interventions against malaria, due to its relevant functional role [29]. This work identifies and characterizes bovipain-2, the *B. bovis *ortholog of falcipain-2. The biological significance of this protein is underscored by the observation that *B. bovis *growth can be inhibited using cysteine-proteinase inhibitors [24].

Based on their sequences, falcipain-2 and bovipain-2 are classifiable as cysteine peptidases belonging to Clan CA, subfamily C1A. This peptidase subfamily is characterized by the presence of four catalytic Q, C, H, and A residues present in three separate, well conserved regions of the primary sequence that corresponds to the mature protease, which are known as the eukaryotic thiol (cysteine) proteases cysteine, histidine, and asparagine active site regions (Figure 2). In the final tertiary conformation of the protein, the catalytic amino acids are brought together and constitute the enzyme active site. Importantly, predicted models of the putative catalytic site in falcipain-2 and bovipain-2 showed a similar arrangement of the relevant C, H, and N regions that form the active site of the enzyme in both proteins, despite high sequence polymorphisms in the intervening regions (data not shown). Furthermore, alignment of bovipain-2 orthologs from different apicomplexan parasites showed strict conservation of the catalytic residues and low polymorphism in their surrounding areas (Figure 2). Additionally, full sequence conservation was observed among bovipain-2 protein sequences of *B. bovis *geographical isolates in the whole region that harbors the active site. This constraint for genetic variation is likely due to the need for keeping the structural conformation of the protein in order to preserve its activity. Thus, taken together these data support a cysteine protease function for bovipain-2, similar to what was previously described for falcipain-2.

Interestingly, apart from bovipain-2, only 3 other C1 cysteine proteinase encoding genes seem to be present in the *B. bovis*-genome. The bovipain-2 encoding gene corresponds to gene families in *T. annulata *and *T. parva *of 7 and 6 members, respectively. In contrast, the other cysteine proteinase encoding genes of *B. bovis *have a single ortholog equivalent in *T. parva *and *T. annulata*. We hypothesize that the expanded cysteine-protease family of bovipain-2 type may have a specific function in *T. parva *and *T. annulata *associated with the additional schizont parasite stage and more complex life cycle of *Theileria *sp. parasites. However, this notion would need to be demonstrated in future investigations. Gene duplications and tandem arrays of similar isoforms of clan CA peptidases have also been found in other related protozoan parasites, but the biological implications of this phenomenon remain unclear [30].

In *P. falciparum *falcipain-2 localizes in the food vacuole, where hemoglobin digestion takes place [31]. However, falcipain-2 expression was detected also outside of the food vacuole and near the erythrocyte membrane skeleton which is consistent with the proposed involvement of erythrocyte membrane skeletal protein cleavages in merozoite egress [15]. The punctate immunofluorescence pattern shown in Figure 6 indicates that, similar to its *P. falciparum *ortholog, bovipain-2 also appears to be localized in a yet undefined, internal organelle of the parasite. Food vacuoles have been described in *Babesia sp*. but only on the basis of morphological evidence [32,33]. Thus, further investigations need to be carried out to more exactly elucidate the intracellular location of bovipain-2. However, similarly to *Plasmodium*, anti-bovipain-2 antibodies also reacted with the cytoplasm of *B. bovis*-infected erythrocytes of different strains tested, while smears of non-infected erythrocytes showed no reactivity. These observations suggest first, that bovipain-2 and erythrocyte proteases do not share cross-reactive B-cell epitopes and also, that bovipain-2 is released into the erythrocyte cytoplasm. Occasionally, some erythrocytes in which no parasites could be detected showed cytoplasmic reactivity in smears of *B. bovis*-infected erythrocytes (data not shown). Since non-infected erythrocyte smears showed no reactivity with anti-bovipain-2 antibodies, a possible explanation for this observation is that the parasites egressed from the reactive erythrocytes before the smears were prepared, leaving released proteins behind. Reactivity towards empty erythrocytes of antibodies that recognize babesial proteins was previously observed in the case of the merozoite spherical body protein, Bb-1, which can be found on the cytoplasmic side of the erythrocyte membrane [34]. It has been suggested that Bb-1 is secreted by *B. bovis *into the erythrocyte cytoplasm, where it might be involved in invasion and/or exit processes.

Anti-bovipain-2 antibodies cross-reacted with *B. bigemina *merozoites. A 48.5% identity in the predicted amino acid sequences of bovipain-2 and a putative *B. bigemina *protein named babesipain [35,36] (Table 2) could be responsible for the observed cross-reactivity. The cytoplasm of *B. bigemina*-infected erythrocytes also reacted with anti-bovipain-2 antibodies, suggesting that release of babesipain from *B. bigemina *merozoites has also taken place.

Expression of cysteine proteases in cultured, wild type or attenuated *B. bovis *parasites can be affected by distinct possible selective pressures. This is significant since the pattern and level of expression of cysteine proteases were also regarded as potential factors affecting the virulence of parasites [18]. Sera from bovines experimentally infected either with the BboS2P or BboR1A Argentinean strains reacted with the recombinant bovipain-2, confirming *in vivo *expression and immunogenicity of the protein in the blood stages of the parasites regardless of their degree of virulence.

However, because four out of six sera from infected animals tested failed to recognize recombinant bovipain-2, the results also suggest that bovipain-2 might be poorly immunogenic during acute infection. It has been reported that subdominant antigens may prove to be more effective as vaccine candidates than immunodominant antigens [37]. Based on this supposition, bovipain-2 constitutes an interesting candidate for subunit vaccine development. Alternatively, it is possible that these sera also contain antibodies reactive with conformational epitopes in the native protein that are not able to recognize the recombinant version of bovipain-2.

*In silico *analysis of the *B. bovis *predicted proteome using the MEROPS database has shown the presence of 66 proteases, that belong to the cysteine (n = 18), serine (n = 18), metallo (n = 19), threonine (n = 6) and aspartic (n = 5) classes (manuscript in preparation). Although this number is small compared to *P. falciparum *(n = 93), the high number of protease-encoding genes appears to indicate that these enzymes participate in critical metabolic processes and/or signalling mechanisms that need to be separately regulated. On-going experiments to analyze which of these proteases are transcribed in the merozoite and other stages of the parasite life cycle will throw light on their possible functional roles and their suitability as targets for improved control methods for bovine babesiosis.

## Conclusions

Collectively, the data presented in this study identifies and demonstrates the *in vitro *and *in vivo *expression of bovipain-2, a cysteine protease, in *Babesia bovis*. Because this family of proteases plays significant roles in the biology of related parasites, they might become additional targets for improving the control of bovine babesiosis. Whether antibodies against bovipain-2 present during natural infection reduce the parasite burden and whether this protein is a viable candidate for vaccine development or for new anti-babesial drugs will be the subject of future research.

## Methods

### Bioinformatic analysis

C1 cysteine peptidases were identified from the proteome as predicted by the genome of *Babesia bovis *(T2Bo strain), *Theileria annulata *(Ankara strain) and *T. parva *(Mumuga strain) [38] using the MEROPS database [6,39]. Orthology was established when the complete genome sequence of the corresponding organism was available using reciprocal BLASTP best hit [40]. Percentages of protein identity were calculated using a BLOSUM62 matrix. Possible deleterious effects of amino acid substitutions on protein function were predicted by SIFT [41,42]; which takes into account sequence homology and the physical properties of amino acids. After alignment of the amino acid sequences of the catalytic region of cysteine proteinases of *B. bovis*, *T. annulata *and *T. parva *by clustalw [43], their phylogenetic relationship was constructed by neighbor joining using MEGA4 [44,45]. Synteny studies were carried out with GEvo [46,47]. Parameter settings using BLASTZ were as follows: word size 8, gap opening penalty 400, gap extension penalty 30, and score threshold 1800. The minimum high scoring sequence pair (HSP) length for overlapped features was set to 50. For the prediction of glycosylation, NetNGlyc and NetOGlyc were used [48-50].

### Samples and DNA extraction

The following *B. bovis *strains and isolates were used: BboR1A, an Argentine vaccinial strain [51]; BboS2P and BboM3P, pathogenic isolates derived from clinical cases in Salta and Corrientes, Argentina, respectively; Veracruz, a pathogenic isolate from Mexico [52]; Brazil and Uruguay, which originated from clinical cases from these countries.

BboR1A and BboS2P strains were maintained in *in vitro *culture on bovine erythrocytes in M199 medium, supplemented with 40% bovine serum, at 37°C, basically as described by Levy and Ristic [53] but using a regular 10% CO_2 _atmosphere. BboM3P, Brazil, Uruguay and Veracruz isolates were amplified in splenectomized calves. *In vitro *cultured merozoites were partially purified by differential centrifugation as previously described [54]. In the case of parasites amplified in splenectomized calves, whole blood was centrifuged (3000 × g, 30 min, 4°C), the pellet was frozen at -20°C overnight, and then washed thrice with PBS by centrifugation (3000 × g, 30 min, 4°C) to remove released hemoglobin. DNA was purified from both kinds of samples with phenol/chloroform/isoamylic alcohol (Sigma-Aldrich, St. Louis, MO) extraction and ethanol precipitation, using established procedures [55], followed by RNAse (USB, Cleveland, OH; 20 μg/ml, 45 min, 45°C) and proteinase K (USB; 100 μg/ml, overnight, 45°C) treatment. DNA samples were re-extracted, dissolved in distilled water, quantified by spectrophotometry, and kept at -20°C until use.

### PCR amplification

An aliquot of DNA (50 ng) was added to a PCR mix (25 μl final volume) containing 2 mM Cl_2_Mg, 200 μM each dNTP, 1 U Taq polymerase (Invitrogen) and 0.4 μM bovipain-F and bovipain-R primers (5'-ATGGAAATACCGGCTGCTG-3' and 5'-ATATGGGACATAACCGTAAG-3', respectively). After 10 min incubation at 95°C, samples were subjected to 35 cycles of: 60 s at 95°C, 45 s at 55°C and 60 s at 72°C, followed by an extension period of 10 min at 72°C. Amplicons were visualized in ethidium bromide-stained 0.8% agarose gels and sized by comparison with a 1 kb Plus DNA ladder (Invitrogen).

### RNA extraction

Total RNA was extracted from 5 × 10^6 ^infected erythrocytes of *in vitro *cultured *B. bovis *BboS2P and BboR1A parasites, using the NucleoSpin RNAII kit (Macherey-Nagel, Germany), according to the manufacturer's instructions. RNA integrity was protected with 1 U/ul of Riboblock (Fermentas, Burlington, Canada). Samples were then treated with DNAse (Invitrogen) for 15 min at room temperature, followed by addition of 2 mM EDTA and incubation at 65°C for 10 min for DNAse inactivation. The final samples were kept at -80°C until use.

### RT-PCR

RNA aliquots (150 ng) from either the BboS2P or the BboR1A strain of *B. bovis *were incubated with SuperScript III One-Step RT-PCR with Platinum Taq DNA Polymerase (Invitrogen), 2× reaction mix (following the manufacturer's protocol), and 0.4 μM of bovipain-F and bovipain-R primers in a final reaction volumen of 25 μl. Parallel negative controls were prepared, containing the same composition without Reverse Transcriptase. As positive control, primers that amplify the MSA-2c transcript [28] were used with the same RNA samples. All samples were incubated for 30 min at 55°C, followed by 2 min at 94°C and then subjected to 40 cycles of 45 s at 94°C, 45 s at 55°C and 60 s at 68°C, with a final extension period of 5 min at 68°C. Amplicons were visualized and sized as described above.

### Sequencing

PCR and RT-PCR amplicons were purified using the GFX PCR DNA and Gel Band Purification kit (GE Healthcare), and both strands were sequenced using specific primers, at the Internal Service of Genotyping and Sequencing Ibiotec (Institute of Biotechnology, CICVyA, INTA-Castelar, Argentina). The Big Dye Terminator v3.1 kit (Applied Biosystems, Carlsbad, CA, USA) and a 3130xl Genetic Analyzer equipment (Applied Biosystems) were employed.

### Expression and purification of recombinant bovipain-2

The entire *bovipain-2 *ORF, amplified by PCR from DNA of *B. bovis *BboR1A was cloned into pCR2.1 vector (Invitrogen). The correct reading frame of the recombinant construct was verified by sequencing. After digestion of bovipain-2-pCR2.1 with *EcoR*I (Promega, Madison, WI), the restriction fragment was purified using GFX PCR DNA and Gel Band Purification kit (GE Healthcare) and cloned in-frame in the prokaryotic expression vector pRSETB (Invitrogen). *E. coli *Rosetta (DE3, Novagen) cells were transformed with the recombinant vector and expression was induced by overnight exposure to 1 mM IPTG (Invitrogen) in Luria Broth medium containing 50 μg/ml ampicillin and 35 μg/ml chloramphenicol (Sigma-Aldrich). Cells were pelleted by centrifugation and the pellet was solubilized in Laemmli buffer. Aliquots of this lysate were electrophoresed in preparative 12% polyacrylamide minigels in the presence of SDS. A protein molecular weight marker (Fermentas) was run in parallel to size the bands. Gel sections containing 50-55 kDa proteins were excised, and placed in a cellulose membrane dialysis tubing retaining proteins of molecular weight of 12 kDa or greater (Sigma-Aldrich). Proteins were electroeluted in a horizontal electrophoresis chamber at 35 mA for 2.5 h in SDS-PAGE running buffer, followed by dialysis against PBS.

For Western blots, samples were 5× concentrated inside the dialysis membranes by exposure to solid sucrose at room temperature, followed by dialysis against PBS. Finally, 1 mM PMSF, final concentration (Calbiochem, San Diego, CA) was added, protein concentration was measured by the Micro BCA Protein Assay Kit (Pierce, Rockford, USA), and samples were stored at -80°C until use.

### Production of antisera

Adult male Balb-c mice (n = 2) were inoculated with bovipain-2, produced and partially purified by electroelution as described above, emulsified in Sigma Adjuvant System (Sigma-Aldrich). Inoculations took place at days 0, 20, 40 and 55 and each consisted in 200 μl emulsion, containing 50 μg of protein, which were administered in several spots through the subcutaneous and intraperitoneal routes. Hyperimmune serum aliquots were collected by heart puncture at day 70 p.i. after euthanizing the mice with CO_2_.

### Detection of bovipain-2 expression in *in vitro *cultured merozoites

*In vitro*-cultured *B. bovis *BboS2P merozoites were purified by centrifugation in Percoll gradients as described before [54]. Samples containing 7 μg of protein were dissolved in Laemmli buffer, electrophoresed in 10% polyacrylamide minigels in the presence of SDS, and transferred to nitrocellulose membranes. A protein molecular weight standard from Fermentas was run in the same gels. Blots were blocked with 10% (w/v) dry low-fat milk in PBS/0.01% Tween-20 (PBST) and incubated with a pool of mouse anti-bovipain-2 hyperimmune serum (day 70 p.i.), or with a pool of serum obtained at day 0 from the same mice (1/50 dilution in blocking buffer). After three washes with PBST, reactivity of antibodies with merozoite proteins was detected by incubation with peroxidase-labeled anti-mouse IgG (Santa Cruz Biotechnologies, CA) followed by ECL reagent (GE Healthcare). Chemiluminiscence was visualized on Hyperfilm sheets (GE Healthcare) developed with Kodak photographic reagents (Rochester, NY).

### Reactivity against bovipain-2 of sera from *B. bovis*-infected cattle

Partially purified recombinant bovipain-2 obtained as described above was electrophoresed in 12% polyacrylamide gels in the presence of SDS, followed by transfer to nitrocellulose membranes, blocking as above, and incubation with the following sera: a) monoclonal anti-histidine antibody (GE Healthcare); b) bovine sera negative to *B. bovis *antibodies from a tick-free region of Argentina (n = 2); or c) sera of bovines experimentally infected with *B. bovis *BboR1A (n = 3) or BboS2P (n = 3), (day 63 p.i.). Sera from groups (b) and (c) were pre-incubated with an *E. coli *lysate (0.1 mg/ml, 37°C, 1 h). Bovine sera were tested in 1/10 dilution. Blot (a) was incubated with peroxidase-labeled anti-mouse IgG, and blots (b) and (c) with peroxidase-labeled anti-bovine IgG (Santa Cruz Biotechnologies) and the reactions were visualized by ECL chemiluminiscence. As positive control, recombinant *B. bovis *MSA-2c [28] was used.

### Indirect immunofluorescence assay

Aliquots (5 ml) of *in vitro *cultured suspensions of *B. bovis *merozoites in bovine erythrocytes (10-15% infected erythrocytes) from the strains BboS2P (Argentina) or RAD (Mexico); or with *in vitro *cultures of *B. bigemina*, strains BbiS2P or Mexico, were washed thrice with PBS by centrifugation and resuspended in PBS/0.5% normal horse serum. Smears of these suspensions or of non infected erythrocytes (5 μl) were prepared, divided in sections with Enamel paint and incubated with: a) normal mouse serum in a 1/60 dilution; b) a pool of two anti-bovipain-2 mouse sera (70 dpi) in the same dilution; and c) anti-RAP-1 23/70.174 Mab [27], 8 μg/ml. The fixed immunofluorescence assay was carried out as described before [56], using fluorescein isothiocyanate (FITC)-labeled goat anti-mouse IgG (Dacko, Glostrup, Denmark), or donkey anti-mouse Alexa Fluor 488 (Molecular Probes, Invitrogen, Eugene, OK). Slides were mounted with glycerol/PBS (1:1, v/v) and observed under fluorescence microscopy (400 and 1000 × magnification).

## List of abbreviations

PMSF: phenylmethylsulfonyl fluoride; TLCK: N-α-p-tosyl-L-lysine chloromethyl ketone; TPCK: L-1-tosylamide-2-phenylethyl-chloromethyl ketone; Q: glutamine; C: cysteine; H: histidine; A: alanine; aa: amino acid.

## Competing interests

The authors declare that they have no competing interests.

## Authors' contributions

MM designed the study, took care of most of the experimental aspects of this work and was in charge of drafting the manuscript. IE carried out the *in vitro *culture of *B. bovis *parasites and participated in the experiments on the expression of bovipain-2 in merozoites. MD carried out the extraction of *B. bovis *DNA and participated in the experiments of immune recognition of bovipain-2 by bovine antibodies. JJM carried out the immunofluorescence assays on Mexican *B. bovis *and *B. bigemina *strains. CES took care of the localization of the bovipain-2 gene in the *B. bovis *genome and helped to draft the manuscript. LS designed the bioinformatic approaches of the work, took care of the construction of the phylogenetic tree and synteny studies and helped to draft the manuscript. MFC conceived of the study, participated in its design and coordination and helped to draft the manuscript. All authors read and approved the final manuscript.
